# Painless Obstructive Jaundice Secondary to a Common Bile Duct Abscess: A Delayed Sequela of Cholecystectomy

**DOI:** 10.1155/2009/628197

**Published:** 2009-12-21

**Authors:** Katherine Fairhurst, Andrew Strickland, Franklin H. G. Bridgewater, Guy J. Maddern

**Affiliations:** The Queen Elizabeth Hospital, University of Adelaide Discipline of Surgery, Woodville, South Australia, SA 5011, Australia

## Abstract

Complications related to cholecystectomy are well described. Most occur in the early postoperative period and are recognised either at the time of, or shortly after surgery. Clinical sequelae occurring years following cholecystectomy are rare and infrequently reported. In addition, most delayed complications are related to the continuing presence or new formation of gallstones. In this paper we present a unique case of an abscess of the common bile duct wall, presenting with painless obstructive jaundice more than 30 years following an open cholecystectomy, *without* the presence of gallstones. The clinical presentation, investigations, and treatment are discussed with a review of other relevant reported cases in the literature.

## 1. Introduction

Commonly reported complications of cholecystectomy include damage to the common bile duct [1], bile leaks from the gallbladder bed or cystic duct stump [2], dropped gallstones [3], and damage to other structures such as the small bowel, liver, or diaphragm [4]. The complications of laparoscopic surgery have lessened with growing technical expertise, and laparoscopic cholecystectomy is now considered the gold standard [5]. However, the essential steps of a cholecystectomy remain the same. These steps include adequate visualisation of the structures to obtain “the critical view,” secure occlusion of the cystic duct and artery, good haemostasis, and the treatment of any bile leaks from the gallbladder bed, or bile or stone spillages from an iatrogenically perforated gallbladder. Inadequate performance of any of these steps can lead to complications many years following the initial operation, as presented in this paper, and as such attention to operative detail is of paramount importance.

## 2. Case Report

A 70-year-old female presented to a rural hospital with painless jaundice. She had undergone a routine open cholecystectomy 30 years earlier at the same hospital. Biochemical markers showed an obstructive pattern: Bilirubin 155 *μ*mol/L (6–24), ALP 262 U/L (30–110), ALT 272 U/L (0–55), AST 161 U/L (0–45). The white cell count was within the normal range at 6.65 × 10^9^/L (4–11), and the CRP was mildly elevated at 11 mg/L (<8.0). In the 30-year interval, she had experienced no other episodes of jaundice, abdominal pain, or unexplained fever and had undergone no other abdominal surgery. Unfortunately the hospital records (on microfilm) were incomplete so no operation note from the cholecystectomy 30 years previously was available. The anaesthetic chart and drug chart were retrieved and revealed that no antibiotics were given and no specific intraoperative difficulty or postoperative complications were recorded in the clinical records. The patient also confirmed an uneventful recovery.

**Figure 1:**
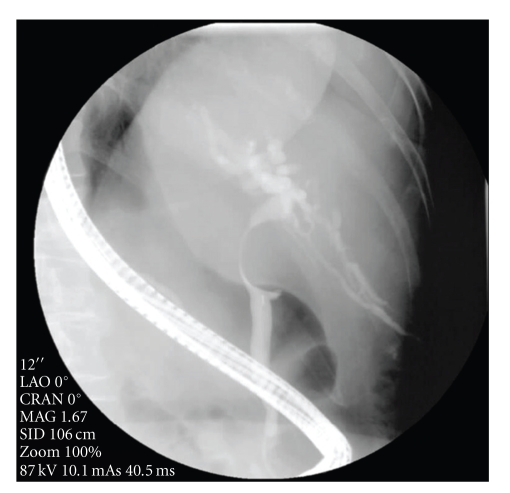
Stricture of the proximal bile duct at ERCP.

She was referred to the gastroenterologists at a tertiary centre for endoscopic retrograde cholangiopancreatography (ERCP). The images at ERCP demonstrated a round filling defect approximately 2 cm in diameter associated with a smooth stricture consistent with extrinsic compression in the proximal bile duct (Figure 1). The common bile duct was cannulated without difficulty and measured 8 mm in diameter. A guidewire was easily placed past the area of compression and insertion of a 10 French; 12 cm Cotton Leung biliary stent was performed. The jaundice resolved within 72 hours of the procedure. No antibiotics were given at the time of the ERCP. No malignant cells were identified from the aspirated bile sent for histology. The patient was discharged the day following the ERCP and referral to the hepatopancreaticobiliary surgical team was made for further investigation into the cause of the jaundice.

**Figure 2:**
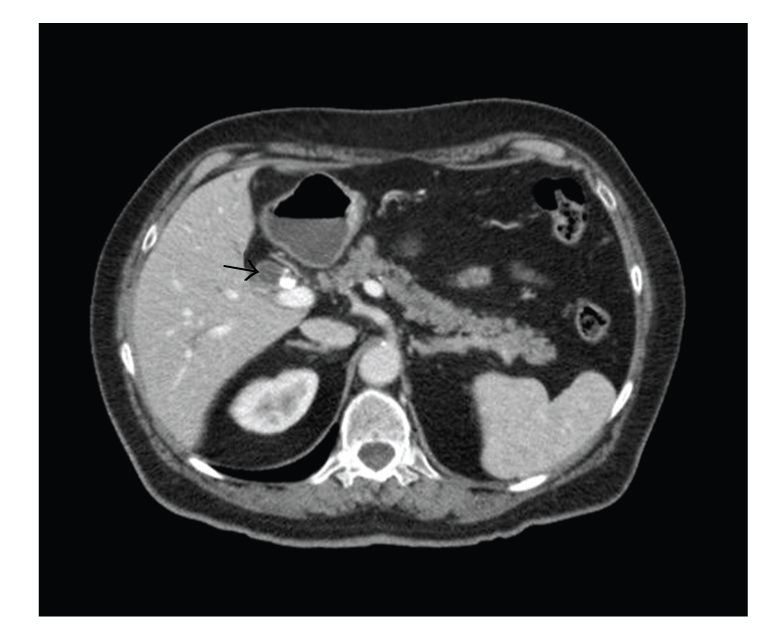
CT demonstrating lesion arising from common bile duct (arrow) with the common bile duct stent seen more medially.

CT imaging demonstrated mild intrahepatic dilatation. No lesions other than a rounded soft tissue mass immediately lateral to the stent and below the porta hepatis were identified (Figure 2). This was consistent with the ERCP findings. The mass measured slightly smaller on the CT images at approximately 1.5 cm in diameter. The mass had varying density but seemed complex in nature and as such the possibility of a choledochal cyst was raised. Diagnostic laparoscopy was performed one month following the ERCP. The view was obscured by adhesions from the previous open cholecystectomy completed via a right paramedian incision. No antibiotics were given at the time of the laparoscopy. An esophagogastroduodenoscopy and colonoscopy excluded primary malignant lesions in those areas. Following extensive discussion at the hepatopancreaticobiliary multidisciplinary meeting and with the patient, it was decided that due to the diagnostic uncertainty of the obstructing tumour in the hepatic duct, laparotomy and attempted excision of the lesion should be performed.

At operation, the common bile duct was identified and the lesion located within the proximal bile duct at the usual position of the insertion of the cystic duct, though the cystic duct stump itself could not be identified. The dimensions of the lesion were similar to those measured on the CT images and the common bile duct again measured 8 mm in diameter. During dissection pus started exuding from the duct wall. A sample was sent for microbiological analysis. No bile was seen in the purulent discharge and probing of the underlying cavity did not reveal a communication with the common bile duct. Because of the possibility of malignancy, the common bile duct was opened and choledochoscopy showed normal primary and secondary bifurcations of the common bile duct, a normal duct distally and no apparent communication with the abscess cavity. The lateral wall of the abscess cavity was resected and sent for histology. This confirmed mild, chronic nonspecific inflammation and fibrosis only. The stent placed at ERCP three months previously was removed and the tip sent for culture. Because the medial wall of the common bile duct was found to be thinned and delicate due to the abscess formation and consequent resection, a T-tube was inserted and sutured into position.

**Figure 3:**
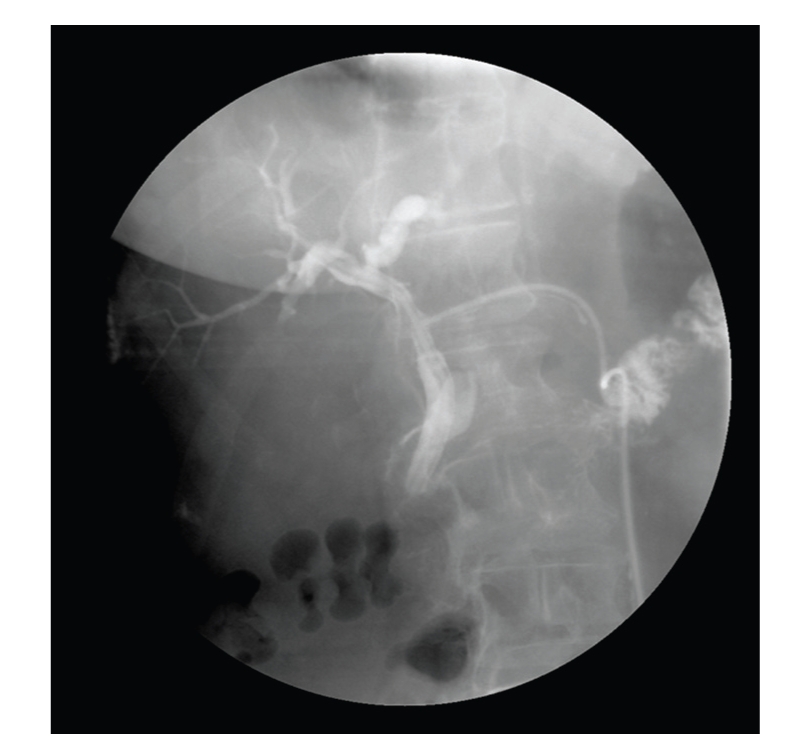
T-tube cholangiogram demonstrating resolution of the stricture.

Intravenous cephazolin and metronidazole were given intraoperatively as part of the hospital microbiology guidelines for antibiotic prophylaxis of biliary tract surgery. These antibiotics were given for 4 days postoperatively with the addition of gentamycin, whilst culture results were awaited. *Klebsiella ornithinolytica* and *Enterococcus casseliflavus* were subsequently identified and were sensitive to the prescribed antibiotic regime. The postoperative recovery was uneventful with no episodes of sepsis. A T-tube cholangiogram thirteen days postoperatively showed no abnormalities (Figure 3). The tube was clamped following the cholangiogram and removed without incident two weeks later.

## 3. Discussion

Complications relating to cholecystectomy have been extensively documented throughout the literature. The diagnostic difficulties associated with nonmalignant, biliary strictures such as those caused by autoimmune processes, have also been discussed [6]. This case of an abscess forming within the wall of the common bile duct presenting as obstructive painless jaundice more than 30 years following this procedure and without the presence of gallstones, appears to be unique in the literature to date. The most logical explanation for this outcome is that of a small leak from the cystic duct stump with the formation of a chronic abscess. The possibility of iatrogenic injury at the time of ERCP is unlikely because the procedure was technically simple and the images at the time of the ERCP demonstrate the presence of the obstructing lesion clearly. Additionally, the procedure was not followed by any episodes of pain or sepsis during the interval between the ERCP and laparotomy; a period of 3 months in total. The abscess became clinically evident only when it finally obstructed the common bile duct resulting in jaundice. Our patient had remained entirely well with no symptoms of sepsis or pain until her presentation with jaundice. It is unclear why she suddenly became jaundiced or what factors may have been involved in reactivating the abscess.

Other cases of abscesses around the common bile duct causing jaundice have been reported previously. Stevens et al. reported a case of obstructive jaundice 12 months following laparoscopic cholecystectomy [7]. ERCP and stenting were performed but the stent rapidly occluded necessitating laparotomy. An abscess cavity containing multiple small gallstones, presumed to have been spilt during the original operation, was found surrounding the common bile duct causing compression and fibrosis. Plehwe and Glenn in 1978 recounted a case of obstructive jaundice on a background of recurrent abdominal pain for one year [8]. Spontaneous perforation of the common bile duct by a one centimetre gallstone causing abscess formation just below the cystic duct and consequent obstructive jaundice was found at open cholecystectomy. Xing et al. described a 73-year-old male presenting with an abscess in the gallbladder fossa 6 years following laparoscopic cholecystectomy [9]. The abscess resolved following CT-guided aspiration but 4 years later ERCP performed for ascending chlolangitis secondary to common duct stones, revealed the presence of a “phantom gallbladder” image. The authors postulate that the cystic stump was communicating with the gallbladder fossa in some way, presumably relating to the previous abscess as a result of fistula formation into the cystic duct. The alternative theory was that increased pressure in the cystic duct stump associated with choledocholithiasis caused rupture into the gallbladder fossa. However, all of these reports are associated with complications from the presence of gallstones specifically. This is in contrast to this particular case.

*Enterococcus casseliflavus* is rarely isolated from clinical specimens but is of specific interest because of its low level resistance to vancomycin and its low virulence exhibited clinically [10]. *Klebsiella ornithinolytica* is one of the least common species of *Klebsiella* and is occasionally isolated from wounds and abscesses but often represents colonisation only. These isolates are of interest in this case as they represent unusual pathogens which may not have been treated at the time of the original surgery and may consequently have contributed to chronic abscess formation.

## 4. Conclusion

The importance of precise identification and adequate occlusion of the cystic duct stump and the prevention of bile leaks at cholecystectomy is demonstrated. The classical teaching of “painless obstructive jaundice; malignant until proven otherwise” is also reiterated. Modern imaging with ERCP and CT and less invasive tests such as diagnostic laparoscopy used here could not prove entirely the nonmalignant nature of this lesion and as such exploratory laparotomy was warranted. This case demonstrates a rare cause of painless obstructive jaundice, and a uniquely reported complication of cholecystectomy in the literature to date.
